# A review of dairy cattle heat stress mitigation in Indonesia

**DOI:** 10.14202/vetworld.2023.1098-1108

**Published:** 2023-05-24

**Authors:** Santiananda Arta Asmarasari, Nurul Azizah, Sutikno Sutikno, Wisri Puastuti, Azhar Amir, Lisa Praharani, Supardi Rusdiana, Cecep Hidayat, Anita Hafid, Diana Andrianita Kusumaningrum, Ferdy Saputra, Chalid Talib, Agustin Herliatika, Mohammad Ikhsan Shiddieqy, Sari Yanti Hayanti

**Affiliations:** 1 Research Center for Animal Husbandry, Research Organization for Agriculture and Food, National Research and Innovation Agency of Indonesia, Cibinong Science Center, Jalan Raya Jakarta – Bogor, Cibinong, Bogor 16915, West Java, Indonesia; 2 Research Center for Applied Zoology, Research Organization for Life Science and Environment, National Research and Innovation Agency of Indonesia, Jl. Raya Jakarta-Bogor Km 46, Cibinong, Bogor, 16911, West Java, Indonesia

**Keywords:** dairy cattle, heat stress, Indonesia, tropical country

## Abstract

Indonesia is a tropical country with a hot climate. In tropical nations such as Indonesia, heat stress is a key reason for the reduced productivity of dairy cattle. Heat stress is a combination of internal and external stimuli that affects an animal, raises its body temperature, and causes it to react physiologically. Most Indonesian dairy cattle are Friesian Holstein (FH), imported from European nations with a temperate environment with low temperatures in the range of 5°C–25°C. Indonesia has a tropical climate with a high ambient temperature that can reach 34°C during the day and the local relative humidity varies between 70% and 90%. Temperature and humidity are two microenvironment factors that may impact the production and heat release in FH cattle. More than 98% of the entire dairy cattle population in Indonesia is found on Java Island. On Java Island, there are between 534.22 and 543.55 thousand heads of cattle, while the dairy cattle population outside Java Island is just 6.59 thousand heads of cattle. The milk output climbs by an average of 3.34% per year, or approximately 909.64 thousand tons and the average annual growth in whole milk consumption was 0.19 L/capita. Indonesian cow milk output has been unable to keep pace with the country’s increasing demand. This study aimed to review the strategies to mitigate heat stress in FH dairy cattle in Indonesia.

## Introduction

Indonesia is a tropical country with hot climate conditions, which causes the performance, production, and reproduction of dairy cattle to be disrupted either directly or indirectly due to decreased feed quality and disease development [1]. This is why the average milk production of Friesian Holstein (FH) dairy cattle in Indonesia (10–12 kg/head/day) [2] is far below the average of FH dairy cattle production, as reported by Herbut *et al*. [3], of 41 kg/head/day. In tropical nations such as Indonesia, heat stress is a key reason for the reduced productivity of dairy cattle. Dairy cattle in tropical areas are subjected to prolonged periods of heat stress, which prevents them from recovering from its harmful effects as rapidly as cattle in temperate settings. A longer hot season and higher relative humidity (RH) are climate factors that are more prevalent in areas that are closer to the equator and, thus, more likely to induce heat stress in cattle [4].

Heat stress is a combination of internal and external stimuli that affect an animal, raise its body temperature, and cause it to react physiologically [5]. Heat stress in dairy cattle can severely impact the animal’s well-being. Cattle may become overheated due to excessive external air temperature, humidity, and solar radiation [4]. Dairy cattle maintain a steady body temperature under heat stress conditions by controlling their thermal energy balance [6]. The heat produced by metabolism (maintenance, activity, growth, lactation, gestation, and feed intake) must equal the heat loss to the environment to maintain a thermal energy balance. This thermal equilibrium cannot be maintained if an animal cannot expel sufficient heat created or absorbed during metabolism [7]. Most of Indonesian dairy cattle are FH, imported from European nations with a temperate environment with low temperatures in the range of 5°C–25°C [1]. Dairy cattle are extremely sensitive to modest climate changes, especially when the temperature and humidity are high. Only in their thermal neutral zone (TNZ) do dairy cattle exhibit their genetic potential to its fullest, with the lowest physiological costs and the highest level of productivity [6]. When an animal does not need to exert energy to maintain a normal body temperature, the temperature range is called the TNZ. Holstein cattle are not well adapted to severe heat stress [8]. Conduction, convection, radiation, and evaporation are some of the ways by that cattle can release heat. However, the difference between the cattle body and environment temperatures determines how successful these heat dissipation techniques are [9]. Maintaining a stable body temperature is referred to as regulating the temperature or heat. When dairy cattle start to feel uneasy, this process begins. In general, the process of thermoregulation entails a thermal balance between the heat produced and the heat released [10]. Livestock work hard and generate heat in their body to provide the energy required to live (activities and environmental adaptation). The heat generated depends on livestock operations and feed consumption, which is expressed as the total digestible nutrient (TDN). This index details whether livestock can digest all the components in their feed [6].

Managing heat stress by dairy cattle in tropical countries such as Indonesia is important to increase milk production and maintain FH dairy cattle productivity according to their genetic potential. Therefore, this paper aimed to review the strategies to mitigate heat stress in FH dairy cattle in Indonesia.

## Friesian Holstein Dairy Cattle Production in Indonesia

According to the Republic of Indonesia’s Ministry of Environment and Forestry, almost all of Indonesia has a tropical climate. The bulk of Indonesia is surrounded by consistently warm waters, and an average surface temperature of 28°C near the coast, 26°C inland and in the mountains (600 m above sea level), and 23°C further up in the mountains (1200 m above sea level) is maintained. Indonesia experiences only minor seasonal temperature fluctuations, and there is little seasonal variation in the number of daylight hours. In Indonesia, rainfall predominates over temperature and air pressure as the main climate component. The local RH varies from 70% to 90%. Higher elevations experience cooler temperatures despite little seasonal or regional change in the air temperature. Generally, temperatures drop by 1°C every 90 m above sea level, with night frost sporadically occurring in high-altitude interior mountain regions. As a tropical country, Indonesia has only two distinct seasons – rainy and dry – both cyclical. There is no discernible spring, summer, fall, or winter. The majority of the country, including the islands of Java and Bali, experiences a dry season from April to October and a wet season from November to March, despite the country’s considerable regional variation. However, due to global warming, the seasons are becoming less predictable [11].

Over 98% of Indonesian dairy cattle population is found on Java Island [12]. On Java Island, there were between 534.22 and 543.55 thousand heads of cattle, while the dairy cattle population outside Java Island was just 6.59 thousand heads of cattle. The milk output climbed by an average of 3.34% per year, or 909.64 thousand tons [1]. The average annual growth in whole milk consumption was 0.19 L/capita, and Indonesian cow milk output has been unable to keep pace with the country’s increasing demand [12].

Friesian Holstein cattle are native to the province of Friesland in the northern region of the Netherlands. The *Bos taurus* breed, which resides in temperate areas in continental Europe, includes FH dairy cows [13]. Friesian Holstein cattle were first introduced to Indonesia in the Pasuruan region of East Java in 1891–1892, and then in the Lembang region of West Java in 1900 [1]. The FH cattle strain produces more milk than other types of dairy cows. The increased elevation of the plains saw an increase in milk output (13.45 ± 3.29 kg/day), while cattle in the lowland regions only produce an average of 10.45 ± 2.64 kg/day[12].

Temperature and humidity are two microenvironment factors that may impact the production and release of heat in FH cattle [14]. In Indonesia, which has a tropical climate with a high ambient temperature that can reach 34°C during the day, people and cattle can become overheated and suffer from heat stress. Dairy cows are subject to heat stress when temperatures and humidity are above normal [15]. The ideal temperature and RH air conditions for FH cattle are 13°C–25°C and 50%–60%, respectively [16]. When the temperature rises above this threshold, physiological and behavioral changes occur in livestock. The ideal environmental conditions for the production performance of FH crossbreed dairy cows are achieved at a temperature of 18.3°C and 55% RH [17].

The considerable amount of water vapor present in the atmosphere has an impact on atmospheric humidity. The temperature humidity index (THI), which combines temperature and humidity, assesses how comfortable the environment is for cattle. A THI value of <72 is the comfortable amount of heat stress from temperature and humidity that dairy cows can tolerate. Livestock experience mild stress when the THI value is between 72 and 79, moderate stress between 80 and 89, and severe stress between 90 and 97 [18]. Heat stress occurs when the heat produced is not equal to the heat released by the animal’s body, and the THI can be used to quantify the possibility of heat stress [19]. A limiting factor of heat stress in humid areas is the comfortable THI of dairy cows below 72 [20]. While in dry areas, the air temperature is the heat stress limiting factor. Climate elements such as temperature and humidity impact dairy cow productivity. In Indonesia, the average daily air temperature ranges from 24°C to 34°C with a humidity of 60%–90% [11].

## Heat Stress Indicators in Dairy Cattle

**Figure-1:**
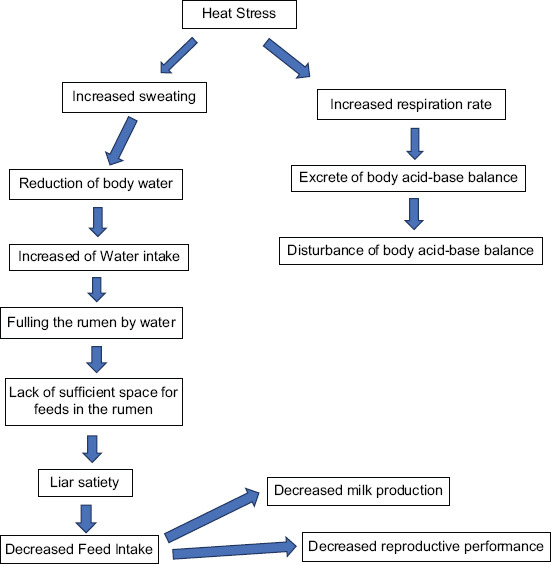
The effect of heat stress on dairy cattle. Source: Atrian and Shahryar [23].

Animal responses to heat stress include reduced feed intake, increased water consumption, metabolic rate changes, increased evaporation losses, blood hormone level changes, and increased core body temperature [21]. Belsare and Pandey [22] reported that heat stress affected several dairy cattle body responses, as presented in Table-1 [22]. Under heat stress, dairy cattle need to drink more water for them to sweat and breathe out heat. The amount of water consumed might rise by as much as 50% when the temperature rises. Meanwhile, Atrian and Shahryar [23] have reported heat stress and reduced milk production stages, as presented in Figure-1 [23]. The following are some of the significant effects of heat stress in dairy cows. Various behavioral cues include seeking protection, refusing to lie down, lack of coordination, and immobility. The breathing rate quickens, heart rate increases, and excessive salivation followed by increased sweating occurs. Dairy cows congregate near water sources and drinking more water causes increased blood flow to their internal organs. There are also some modifications to food digestion, such as decreased or no rumination (chewing feed), and slower feed transit rate.

**Table-1:** The body response of dairy cattle to heat stress.

Indicator	Effect
Body temperature	More than normal
Appetite	Decrease
Pulse rate	Increase
Respiration	Increase
Superficial blood vessels	Dilate
Water from circulatory blood	Added
Muscle tone	Seating
Urinary volume	Decrease
Thyrotropic hormone	Decrease
Thyroxine secretion	Decrease
Adrenaline secretion	Decrease
Male fertility (Semen quality and quantity)	Decrease
Female fertility	Decrease
Conception rate	Decrease
Growth rate	Decrease

Source: Belsare and Pandey [22]

Respiration and heart rate regulation are techniques used by livestock bodies to decrease or release heat received externally. They can be used to determine how dairy cows react to changes in external temperature and humidity [24]. Rectal temperature (body temperature) in dairy cows typically ranges from 38.2°C to 39.10°C. Rectal temperature can be used as an indicator to identify the beginning of heat stress in animals produced by the microenvironment and nutrition [25]. Calves’ and adult dairy cows’ normal respiratory rates vary from 20–40 to 15–35 breaths/min, respectively [26]. As air temperature rises, changes in respiratory rate occur, causing the animals to breathe more frequently to dissipate heat [25]. Several factors impact respiratory rate, including body size, age, physical activity, anxiety, environmental temperature, pregnancy, animal health issues, and animal position [27].

Normal dairy cows have pulse rates that range from 54 to 84 beats/min [28]. The heart rate of livestock rises due to the heat stress induced by extreme ambient conditions. This is due to the increased respiratory rate, which increases respiratory muscle activity and accelerates blood pumping to the skin’s surface and releases body heat. Increased blood temperature directly affects the heart and increases pulse rate. Peripheral vasodilation also has the effect of lowering blood pressure [25].

The ability of livestock to withstand heat in the environment is known as heat tolerance. Animals under heat stress will display changes in body temperature and respiratory frequency [26]. When the heat tolerance coefficient (HTC) = 2, cattle are considered to have a high level of heat resistance; the higher the HTC value, the lower the resistance. This is due to the higher body temperature HTC and higher respiratory frequency. Livestock exposed to heat stress suffers from lowered immunity and compromised physiological processes [29].

Rectal temperature is one indicator to measure heat stress in dairy cattle. Skibiel *et al*. [26] stated that cattle’s rectal temperature varies dramatically from spring (38.36°C) through summer (38.86°C). Rectal temperature is a marker of thermal equilibrium. It can be used to evaluate the challenges that the thermal environment may have on the impact of dairy cow growth, lactation, and reproduction. Most livestock species have lower performance with an increase in rectal temperature of 1°C or less, making body temperature a sensitive marker of physiological response to heat stress in cattle [24]. Because lactating dairy cows are more thermolabile than non-lactating cows. Skibiel *et al*. [26] observed that the rectal temperature of lactating cows was 0.9°C higher than that of prepartum cows under similar environmental conditions. According to Thornton *et al*. [30], a cow’s rectal temperature is 38.5°C under normal conditions and 39.5°C under heat stress conditions. The heat-regulating center, which is located in the hypothalamus, receives temperature-related stimuli and induces physiological changes to heat production or release [31]. The temperature of the body’s organs, both internal and external, is reflected in the core body temperature. Rectal temperature is an indicator of body temperature, whereas skin surface temperature is an indicator of body temperature outside the body [26].

Regarding the skin surface temperature as an indicator, animal skin may serve as a means of heat release through radiation, heat dissipation conduction, evaporation, and convection [32]. The surface temperature of cow skin under desirable microstructure conditions ranges from 33.5°C to 37.1°C [26]. The surface temperature of cattle skin varies according to the amount of heat received. The skin is the body part that is exposed to the most heat and whose temperature directly corresponds to the variations in the surrounding air temperature. The body temperature rises when the environmental temperatures rise, which allows the body to respond to its surroundings. Dairy cows adjust by seeking shade, breathing more quickly, and expanding their blood vessels, which have a small impact on milk output [33]. According to Wankar *et al*. [34], the body temperature recorded by a clinical thermometer only reflects the equilibrium between the heat created and released, not the overall amount of heat generated. When there is an increase in air temperature, it is followed by an increase in heart rate, which is a physiological mechanism in livestock, to increase the frequency of heart rate to dissipate heat. This is an observable reaction of the cow to changes in temperature through an increased heart rate, which is a mechanism of the cow’s body temperature to reduce or release heat received from outside the body [32].

The beginning of heat stress in animals induced by high ambient and air temperatures can be detected by measuring the body temperature. Efferent effector nerves, the hypothalamus, and efferent nerves regulate the body temperature through a feedback process. The hypothalamus serves as the thermostat control center. By regulating the production and emission of heat, the body maintains its internal temperature constant. Dairy cow productivity is impacted by air temperature and humidity because they alter the water, energy, behavioral, and heat balance in their bodies [35]. When it is hot outside, the body begins to sweat and vasodilation, which occurs in substantial volumes, expands the blood vessels, and removes heat from the body. On the other hand, when the temperature is low, the body creates less perspiration and experiences vasoconstriction, which reduces heat loss. Due to its proximity to the weather, the skin strongly correlates with changes in weather and body surface temperature varies depending on the environment’s humidity, the animals’ location (shade), and ventilation [36]. The mechanism of vasodilation is responsible for the process of sweating out heat. Vasodilation occurs when blood vessels enlarge close to the skin surface (the external environment), allowing heat to escape. Because air is a good conductor of heat, the leather bristles are raised to reduce the amount of trapped air on the skin. Variations in the volume of blood flowing through the skin control the amount of heat lost from the body within certain limits. The skin plays a crucial role in receiving hot or cold temperature signals and allows them to reach the pre-optic hypothalamus and central nervous system [37].

The next heat stress indicator for dairy cattle is the respiratory rate. The average respiratory rate in standing dairy cows is 25–65 breaths/min [38]. Meanwhile, Mishra [39] revealed that the average respiratory rate in mature cattle was 10–30 breaths/min. Cows’ response to variations in the surrounding temperature is an increase in the intensity of their respiration rate, which affects the body’s ability to produce more heat. Animals with increased respiratory respiration lose body heat through their respiratory tracts [40].

## Impact of Heat Stress Factors on FH Dairy Cattle in Indonesia and its Comparison to Several Other Countries

Mariana *et al*. [7] reported that FH dairy cattle raised in Indonesia at different altitudes, namely, in the lowlands (temperature 33.1°C; RH 61.8%; THI 83.5), midlands (temperature 29.9°C; RH 57.2%; THI 56.7), and highlands (temperature 25.7°C; RH 56.7%; THI 73.8), resulted in average rectal temperatures of 38.1°C versus 37.9°C versus 37.8°C, skin temperatures of 33.2°C versus 32.6°C versus 32.1°C, body temperatures of 37.4°C versus 37.1°C versus 37.0°C, respiration rates of 44.0 versus 49.9 versus 37.5 breath/min, heart rates of 65.8 versus 72.6 versus 78.8 beat/min, and HTC of 2.9 versus 3.1 versus 2.6, as well as milk production of 7.0 versus 11.3 versus 13.1 kg/day, respectively. It was concluded that the environmental conditions in the lowland areas had a heat stress impact on FH dairy cattle in Indonesia. Meanwhile, Utami and Widiarso [41] reported the physiological responses of FH dairy cattle in Indonesia in the morning (M), afternoon (A), and evening (E) as follows: morning (temperature 22.9°C; RH 83.8%, THI 71.8), afternoon (temperature 34.4°C; RH 42.4%, THI 82.8), and evening (temperature 28.9°C; RH 59.4%, THI 78.2). This resulted in the following effects of heat stress on calves’ heart rate (68.2 (M) vs. 69.1 (A) vs. 66.3 (E) beat/min), respiration rate (38.7 (M) vs. 44.7 (A) vs. 42.1 (E) breath/min), and rectal temperature (38.5°C (M) vs. 39.1°C (A) vs. 38.9°C (E)). The effect of heat stress on the heifers was heart rate (50.5 (M) vs. 59.0 (A) vs. 60.1 (E) beat/min), respiration rate (31.8 (M) vs. 45.5 (A) vs. 44.7 (E) breath/min), and rectal temperature (37.1°C (M) vs. 37.8°C (A) vs. 38.2°C (E)). The effect on lactating cows’ heart rate (63.2 (M) vs. 66.5 (A) vs. 69.2 (E) beat/min), respiration rate (37.4 (M) vs. 51.7 (A) vs. 47.3 (E) breath/min), and rectal temperature (37.6°C (M) vs. 37.7°C (A) vs. 37.6°C (E)). The effect of heat stress on the dry cows was heart rate (79.0 (M) vs. 78.0 (A) vs. 70.7 (E) beat/min), respiration rate (31.0 (M) vs. 49.2 (A) vs. 47.8 (E) breathe/min), and rectal temperature (37.9°C (M) vs. 38.4°C (A) vs. 38.2°C (E)). It was concluded that dairy cows (calf, lactating cow, and dry cows) were exposed to heat stress during the day (morning and evening), whereas heifers did not experience heat stress.

Jaenuddin *et al*. [42] reported a relationship between the altitude and reproductive performance of FH cattle in Indonesia at an altitude of 1200 m above sea level with a temperature of 22.2°C, RH of 90.84%, and THI of 71.22; the cattle had 88.20 days open, 42% conception rate, 1.52 services per conception, and 75.17 days for the first service. Meanwhile, FH dairy cattle raised at 600 m above sea level with a temperature of 26.46°C, RH of 80.41%, and THI of 77.21 had 110.76 days open, 75.76% conception rate, 1.64 services per conception, and 96.42 days for the first service. On the other hand, Tanuwiria *et al*. [43] reported the effect of altitude on the performance of FH cattle raised in West Java Province, Indonesia. It was reported that altitude significantly affected rumination, eating, drinking, lying, and standing. Altitude was divided into groups 1 (<500 m above sea level), group 2 (550–750 m above sea level), and group 3 (>800 m above sea level), resulted in the following effects: rumination (35.46 vs. 39.74 vs. 45.83 times/day), eating (73.82 vs. 108.75 vs. 123.81 min/day), drinking (25.93 vs. 19.18 vs. 18.18 times/day), lying (188.85 vs. 158.04 vs. 117.55 min/day), and standing (49.75 vs. 57.03 vs. 66.73 min/day), respectively. Altitude also significantly affected the thermoregulation of FH cattle in Indonesia, where the altitude groups 1, 2, and 3 resulted in a heat rate (49.46 vs. 47.74 vs. 46.83 beats/min), rectal temperature (40.05 vs. 39.01 vs. 39.02°C), skin surface temperature (41.15 vs. 39.25 vs. 39.15°C), and mammary surface temperature (41.37°C vs. 42.83°C vs. 42.72°C), respectively. This suggests that FH cattle raised in the lowland areas in Indonesia showed symptoms of heat stress.

Setyorini *et al*. [44] reported on the quality and quantity of FH cow milk raised at different altitudes in Indonesia, the highland group with an altitude of 1065 m above sea level, midland 789 m above sea level, and lowland 449 m above sea level. The milk-specific gravity in the highland, midland, and lowland groups for morning milking was 1025 versus 1025 versus 1024 g/mL and for afternoon milking 1024 versus 1024 versus 1022 g/mL, respectively. The resazurin test results were excellent, excellent, and good for the highland, midland, and lowland groups, respectively. The milk production for each group was 13.82 versus 14.59 versus 10.85 kg/head/day. The quality of milk in terms of fat was 3.80 versus 3.98 versus 4.00%, solid non-fat was 8.66% versus 8.51% versus 8.32%, total solids was 12.46% versus 12.49% versus 12.32%, and milk protein was 2.93% versus 2.91% versus 2.73% for the highland, midland, and lowland groups, respectively. The difference in the altitude of the locations of the farms in Indonesia had a considerable effect on the temperature and humidity of the environment. The environmental conditions in lowland areas had a moderate heat stress impact, while the highland and midland areas had a mild heat stress impact on milk-producing cows. The difference in altitude influenced milk production quantity in all three locations, but it did not affect milk quality.

Qisthon *et al*. [45] reported on the physiological conditions of FH dairy cows raised in East Java, Indonesia, with an air temperature of 31.5°C, RH of 72.3%, and THI of 83.6, which showed the following physiological variables: respiration rate of 84 breaths/min, heat rate of 89.1 beats/min, rectal temperature of 39.6°C, HTC of 4.69, dry matter intake of 161 g/BW^0.75^/day, TDN intake of 109 g/BW^0.75^/day, crude protein intake of 27.8 g/BW^0.75^/day, and milk production of 12.4 kg/head/day.

Wicaksana [46] reported that the average rainfall, temperature, and humidity with THI in Lembang, Indonesia (one of the main dairy cattle farming locations in Java island, Indonesia) from January 2011 to December 2017 were as follows: the average rainfall during the rainy season was 245 ± 93.31 mm/month, average temperature of 21.01°C ± 1.82°C, average humidity of 82.44% ± 6.38%, and average THI value of 62.50 ± 1.82 and the average rainfall during the dry season was 131 ± 115 mm/month, average temperature of 20.14°C ± 0.81°C, average humidity of 82.46% ± 7.32%, and average THI value of 61.64 ± 0.82. The average daily milk production during the rainy and dry seasons was 12.68 and 12.89 kg/head/day, respectively. Meanwhile, during the rainy and dry seasons, the calving interval was 15.12 versus 15.01 months, services per conception rate were 2.24 versus 2.22, and empty period was 5.92 versus 5.96 months, respectively.

Mariana *et al*. [47] reported the physiological response and milk quality of FH dairy cattle during a long dry season in the highlands in Indonesia, which was reported to have a THI of 73.9, resulting in the following physiological responses: respiratory rate of 37.50 breaths/min, heart rate of 78.76 beats/min, rectal temperature of 37.94°C, skin temperature of 32.15°C, body temperature of 37.13°C, and milk production of 14.75 kg/head/day. The quality of the milk produced was as follows: dry matter content of 10.19%, milk fat of 2.14%, and milk protein of 2.50%. The high environmental temperature during the long dry season in the highlands of Indonesia caused heat stress. The THI value (73.9) indicated that FH cattle had mild heat stress conditions. The change in the environmental temperature at the end of the long dry season correlated with physiological responses, such as an increase in rectal temperature, body temperature, skin temperature, and heart rate, but not respiratory rate. All animal physiological parameters, except for the heart rate, were within the normal range, but the milk quality produced was lower, indicating that the dairy cattle in the highlands adapted physiologically to the mild heat stress conditions.

A comparison of the effects of heat stress on FH cows reared in Spain was reported by Ramón-Moragues *et al*. [37] using THI (temperature humidity index) _load unit which was calculated for each hour as the accumulated THI values were during the previous 24 h. It was revealed that FH dairy cows reared in an environment without heat stress (THI_load accumulated <1658.76) and those reared in an environment under heat stress (THI_load accumulated >1775.30) exhibited substantial effects on the following parameters (without heat stress vs. under heat stress): eating (8.74 vs. 8.27 min/h); rumination (23.59 vs. 22.12 min/h), rest (16.16 vs. 13.36 min/h), activity (7.58 vs. 7.82 min/h), and heavy breathing (1.50 vs. 5.74 min/h). These results concluded that heat stress affected the behavior of FH cows reared in Spain, namely, heavy breathing, eating, ruminating, resting, and activity. The higher the THI, the less time is available for eating, rumination, and resting.

The report by Imrich *et al*. [48] on the testing the effects of temperature and RH on FH cows in Slovakia revealed that in the summer season (temperature 23°C; RH 66.89%; THI 70.43%) and winter season (temperature 7°C; RH 78.86%; THI 46.16), considerable effects were observed in the following parameters (summer vs. winter): milk yield (49.55 vs. 58.77 kg/head/day); milk fat content (3.71% vs. 3.76%); milk protein (3.44% vs. 3.57%); lactose (5.06% vs. 5.2%); and somatic cells (144,797 vs. 131,500 count/mL). It was concluded that RH and temperature during the summer season in Slovakia have a negative impact on the milk production and composition of cow’s milk. The effect of heat stress caused reduced milk fat, protein, and lactose content, and an increase in somatic cell count.

Micic *et al*. [49] reported the results of a study on the effect of seasons on the performance of FH dairy cows in the Republic of Serbia. It is known that calving season significantly affects daily milk yield, fat content, and protein content, and in the different seasons (winter [temperature −8°C; RH 85%]; spring [temperature 5°C, RH 75%]; summer [temperature 19°C, RH 71%]; and autumn [temperature 14°C, RH 78%]); the parameters of daily milk yield were 18.31 versus 18.69 versus 17.38 versus 16.90 kg/head/day, daily fat content was 3.96% versus 3.94% versus 3.95% versus 3.99%. Daily protein content was 3.22% versus 3.21% versus 3.21% versus 3.22%, respectively. El-Wishy [50] reported the effect of seasons (winter, spring, summer, and autumn) on FH dairy cows in Turkey, showing that the pregnancy rates of Holstein heifers for the seasons of winter, spring, summer, and autumn were 62.6% versus 66% versus 61.5% versus 62%, respectively, while the pregnancy rates of Holstein cows were 39.1% versus 37.2% versus 21.2% versus 24.4%, respectively. Based on the description above, the FH dairy cows in Indonesia and some other countries show heat stress symptoms when raised in an environment with high temperature, RH, and THI.

## Mitigation Strategy to Overcome Heat Stress in Dairy Cattle

An important element that affects dairy cattle productivity is the environment. A dairy cow’s genetic superiority cannot be best exhibited if the environmental conditions are not ideal. The microenvironment is an environmental element that prevents the manifestation of prominent genetic features of animals [51]. Air temperature, humidity, solar radiation, and wind speed are the key environmental elements that provide challenges [13]. If the internal and external elements are within normal ranges and satisfy their needs, dairy cows can live pleasantly and produce optimally. One of the external elements that can influence dairy cows’ comfort and output is the environmental temperature. Heat stress is a primary issue in dairy farming as it can result in financial losses due to decreased productivity [17]. The average worldwide temperature increased between 0.8°C and 1.70°C during the turn of the 19^th^ and 20^th^ centuries and the average global temperature has increased over the past 5 years. As a result, the TNZ and critical temperature of dairy cows have changed [52].

There are several strategies to mitigate the adverse impacts of Indonesia’s climate, namely through management strategy, feeding strategy, and genetic improvement.

### Management strategy

Because heat stress causes more harm to animals than cold stress, the conditions in cow sheds in Indonesia should be adapted to reduce the heat load. The ideal sort of animal shelter is one where the humidity level is between 78.13 and 50.42% and the microenvironment temperature is maintained between 15°C and 25°C [53]. Reduced heat intake and increased heat loss from building animal shelters are the goals of designing an ideal microclimate inside and around the sheds [54]. It is common practice to use water as a cooling agent, either directly on an animal’s body or to chill the microenvironment of the shelter [55]. During the hottest hours of the day, water can be sprayed intermittently or continually on the shelter’s floor and roof to lower their temperature and, in turn, reduce the heat load on the animals [54]. When water is present, grass screens on the sides of the shelter allow the air to move through comfortably, as proper cross-ventilation is necessary. The production efficiency of cows with access to sheds with evaporated coolers was significantly higher than that of cows with access to traditional sheds [55]. More milk was produced in the group kept under a cooled shed than in the traditionally shaded group. By repeatedly applying a small amount of water to the dairy cattle’s bodies at 15–30 min intervals throughout the dry season, their body temperature may be artificially lowered. Water evaporation from dairy cattle is accelerated when fans or blowers were installed. Depending on the RH, skin cooling by 8°C–15°C can be achieved [56].

### Feeding strategy

When feeding animals during hot weather, the following should be kept in mind: feeding frequency, additional feeding times, cooler times of the day, appropriate feeding space, and access to cool water [57]. Reducing the forage-to-concentrate ratio and increased digestibility of the diet can minimize the reduction in milk production [58]. Feeding buffers such as magnesium oxide and sodium bicarbonate enable higher-concentration ratios and can also aid with low-fat milk syndrome [59]. The need for some minerals is increased during hot weather. Overfeeding with highly digestible protein should be avoided during hot weather; the recommended amount is ≤18% [60]. To enhance the amount of energy consumed, additional fat can be added to the feed and overfeeding should be avoided because it affects rumen function. Summer vitamin supplementation offers no additional benefits [61].

For dairy cows under heat stress during mid-lactation, the ideal dietary net energy requirement may vary between 6.83 and 6.92 MJ/kg (1.63–1.65 Mcal/kg) [58]. The feeding of a 1.5% saturated fatty acid supplement was linked to improvements in milk composition regarding the fat, protein, and lactose content and a decrease in rectal temperature during the hottest portion of the day (14:00) [62]. A significant quantity of metabolic heat was saved by actively substituting fermentable carbohydrates with supplementary saturated fatty acids [60]. Milk production increased significantly when dairy cows were fed 200 g of hydrogenated fish fat daily throughout the summer, and the milk protein and fat content improved [63]. Dairy cows under heat stress with supplemented 1.5% diet calcium salts of fatty acids increased milk protein and milk yield per kg of feed intake while lowering the metabolic heat production from 26.4 to 25.1 Mcal/day [64]. Escobosa *et al*. [65] discovered that feeding mid-lactation Holstein dairy cows with 2.54% calcium salts of fatty acids in their diet enhanced the 4% fat-corrected milk and milk fat output during summer.

Lactating dairy cows exhibited substantially reduced feed intake and increased energy maintenance needs when exposed to heat stress [66]. The total mixed ration (TMR) roughage component can be replaced with a more easily digestible NDF of non-roughage origin to enhance the energy input and reduce the energy shortage [3]. High-quality dietary fiber boosts feed intake by enhancing feed digestibility and palatability [59]. Higher milk output, protein, and lactose contents were achieved by feeding heat-stressed dairy cows with beet pulp instead of corn silage by up to 12% of the diet [67]. Feeding Holstein–Friesian dairy cows a TMR plus 27% crushed maize (a type of slowly fermentable grain) reduced the effects of heat stress, as determined by the increased milk production and lower rectal temperature, despite a reduction in milk fat percentage [68]. Feeding with slowly fermentable grains lessens the heat emitted during fermentation and digestion, easing the physiological effects of heat stress and increasing summer milk production in dairy cows [69].

### Genetic improvement

Bertipaglia *et al*. [69] supported the possibility of selecting heat-tolerant traits with the estimation results of genetic variations of some heat resistance properties in cattle, as presented in Table-2 [30, 70, 71]. The selection of heat resistance properties provides benefits in upgrading livestock productivity, although Hansen *et al*. [72] suggest the selection of cows with high productivity in tropical conditions.

**Table-2:** Genetic variation of some properties related to heat resistance.

Parameters	Heritability	Reference
Body temperature	0.40	[70]
Sweating rate	0.22	[70]
Pigmentation	0.11	[70]
Rectal temperature	0.25–0.33	[30]
Coat pigment	0.30	[70]
Coat thickness	0.23	[70]
Coat length	0.08–0.20	[70]
Black coat color	0.20	[71]

Genetic factors influencing heat stress response and heat resistance among cows are numerous, where *Bos indicus* exhibits higher heat resistance than *B. taurus* [72]. In addition, there are differences in response or adaptability to ambient temperature between individual livestock associated with genetic diversity in population livestock breeds that can be used for selection programs. Some research indicates the presence of large genetic variation between enough individuals to allow for heat-tolerant selection to improve the production of cow’s milk fat and protein in Friesian Holstein cattle [51]. The research differentiates milk production from a cow that has no heat stress resistance and milk production from heat-tolerant cattle.

There is genetic control over the body temperature; therefore, selecting traits related to body thermoregulation is an alternative to increasing livestock productivity in tropical areas [32]. In *B. taurus* cows, traits related to body temperature such as body perspiration, skin structure, and color have good heritability and selection based on these traits is effective. Another study by Thornton *et al*. [30] proved the existence of genetic variation in rectal temperature and the negative correlation between rectal temperature and fertility; thus, low rectal temperature selection can increase fertility. However, the selection of rectal temperature indirectly decreases milk production due to the negative relationship between the level of milk production and heat stress resistance. Cows with low rectal temperature will be less resistant to the resulting heat stress and decreased milk production; therefore, this study suggests the selection of cows with high milk production in the tropics [4].

Coat characteristics affect the transfer of heat energy from the body’s skin coat to the environment and the regulation of body temperature [70]. When cattle are exposed to sunlight, the temperature increases between the skin and coat. In a tropical environment, wet, short, thin, and lacking shine provide good heat and water vapor conductivity through the skin layer [73]. Increasing the genetic quality of dairy cows in wet tropical environments through trait selection of short, thin, and shiny coats is important to increase the reproductive efficiency of Friesian Holstein cows.

Several studies have reported the selective production of cattle reared under hot environmental stress. The selection of cattle growth in tropical conditions has shiny skin to allow for setting heat/sweat and more efficient body cooling [74]. Cattle with a smooth and shining coat skin structure has resistance to radiation in the sun and livestock that have short glossy hair are particularly suitable for tropical climate areas compared to cattle with thick and long wooly coats [75]. Cattle with short shiny coats reflect 30%–50% of sunlight, whereas those with thick and long wooly coats only reflect 10% of sunlight [76], making their growth slower. Other research has shown that the density of the hair of the skin is calculated based on the number of hairs per unit area which affects the resistance of the skin surface to ectoparasites in cattle in the tropics [77].

The relationship between the animal skin and coat ambient temperature is closely related to body temperature regulation, affecting livestock productivity. This coat variation is influenced by non-genetic factors such as region (tropical and temperate), cattle breed, age, season, cow health, and feed [78]. Research conducted by Olson *et al*. [79] describes an opportunity to overcome heat stress where a single dominant gene was found to govern the length of body hair and was related to regulating body temperature during heat stress in cattle. If the length of body hair can also influence thermoregulation in dairy cows, introducing this gene would be especially useful in Friesian Holstein cattle reared in tropical area. However, research by Da Silva *et al*. [70] reported that the genetic correlation between Friesian Holstein cows with long coats and milk production was 0.56.

The amount of cattle body heat from radiation sunlight absorbed by the animal’s body is related to the body surface of the animal exposed to direct sunlight. The color of the cattle’s coat is directly related to the amount of solar radiation absorbed by the livestock [32]. The absorption and reflection of sunlight by cattle hair vary greatly between nations and within the same cattle breed [80]. Skin color affects heat stress related to the amount of heat absorbed by solar radiation. A cow that has a black coat will absorb 92%–100% more heat than a cow that has a white coat. Livestock physiologists have long observed the effects of heat and sunlight on production and livestock reproduction related to coat color.

Several studies have reported the effect of coat color on milk production and cattle reproduction (gestation and distance between pregnancies) raised in area tropical climates with high solar radiation [32, 35]. Milk production in hairy dairy cows with predominantly white coats was 275 kg higher than in cows with predominantly black coats. Bos indicus, with a 16% darker coat than brown cows and 58% darker than white cattle, efficiently absorbs and retains body heat [32]. *Bos taurus* is darker in color, which conducts heat, resulting in a higher body temperature, and thereby reducing growth performance, body weight, and milk production compared to a white cow. Research in Florida showed that Friesian Holstein dairy cows with a white coat color covering more than 70% of the body and with or without provided shelter had a lower body temperature and higher production than cows with a black coat color covering more than 70% of the body maintained under the same conditions [72]. The composition and percentage of the white coat color also affected the reproduction of female cattle. Friesian Holstein dairy cows with a greater than 60% white coat color had a higher percentage of pregnancies and the number of empty days after giving birth to the return of gestation was shorter [81]. Therefore, coat color in dairy cows is an important economic property as it can directly affect milk production and reproduction.

In Friesian Holstein dairy cattle color proportions, black leather has moderate to high heritability [71]. The selection of coat color is particularly beneficial for dairy cows kept in the tropics using the ranching system where the radiation intensity of the sun is high. There are variations in important genetic traits that regulate this trait. This thermoregulation shows the potential selection of livestock expected to pass on this trait to their progeny. However, the genetic correlation between heat resistance and milk production is −0.30 [51], which indicates that the selection of milk production that is not followed by the selection of heat resistance will reduce the heat resistance of cows. The genetic correlation of the percentage of a white coat color with the milk production, milk fat, and fat percentage, is −0.12, 0.41, and 0.38, respectively [71]. Nonetheless, this magnitude of the value of genetic correlation is classified as low, and therefore, the combination of selection for heat resistance and milk production is feasible. Further, research is required to determine the genetic potential of heat resistance and selection to increase milk production.

## Conclusion

As a tropical country, Indonesia faces challenges in developing dairy cattle due to the hot temperatures, which causes the dairy cows to experience heat stress. Heat stress is the main factor in the reduced milk production in dairy cows. This review provides insight into how heat stress affects milk production and explains the mechanisms through which milk production is reduced due to heat stress. Three strategies are reviewed in this review, namely, management strategies, feeding strategies, and genetic selection.

## Authors’ Contributions

SAA, NA, SS, WP, AA, LP, SR, CH, AH, DAK, FS, CT, AH, MIS, and SYH: All authors have an equal contribution starting from determining the topic, making an outline, searching for articles, and writing the manuscript. All authors have read and approved the final manuscript.
